# When Rhythm Control Backfires: A Case of Severe QT Prolongation and Polymorphic Ventricular Tachycardia Following Dual Antiarrhythmic Therapy

**DOI:** 10.7759/cureus.108395

**Published:** 2026-05-06

**Authors:** Sachin Sapkota, Abirami Balasubramanian, Prakash Vishakh, Lela Ruck, Tariq Siddiqui

**Affiliations:** 1 Internal Medicine, Texas Tech University Health Sciences Center El Paso, El Paso, USA; 2 Interventional Cardiology, Center of the Heart - A Providence Medical Partners Practice, El Paso, USA

**Keywords:** amiodarone, cardiac pacemaker, qt interval prolongation, sotalol, torsade de pointes

## Abstract

Class III antiarrhythmic agents are widely used for rhythm control in atrial fibrillation, but their proarrhythmic potential, particularly when combined, poses significant clinical risk. We report a case of life-threatening QT prolongation and polymorphic ventricular tachycardia resulting from concurrent sotalol and amiodarone therapy without appropriate washout.

An 88-year-old female patient on chronic sotalol therapy (80 mg twice daily) presented with atrial fibrillation with rapid ventricular response. Intravenous amiodarone was administered without sotalol washout, resulting in severe QT prolongation (QTc 626 ms), deep T-wave inversions, and non-sustained ventricular tachycardia (NSVT). The patient was successfully managed with overdrive pacing, increasing the heart rate from 60 to 80 beats per minute, which suppressed the ventricular arrhythmias and prevented progression to torsades de pointes.

This case underscores the critical importance of recognizing drug-drug interactions between class III antiarrhythmic agents and the necessity of appropriate washout periods. Overdrive pacing remains an effective temporizing measure for drug-induced torsades de pointes. Clinicians must be highly cautious while using multiple QT-prolonging agents, particularly in elderly patients with multiple comorbidities.

## Introduction

Class III antiarrhythmic agents, including sotalol and amiodarone, are cornerstone therapies for maintaining sinus rhythm by prolonging cardiac repolarization through potassium channel blockade [1,2]. However, this mechanism inherently carries proarrhythmic risk, particularly QT interval prolongation and the potentially fatal polymorphic ventricular tachycardia known as torsades de pointes (TdP) [1]. The concurrent use of multiple QT-prolonging antiarrhythmic drugs without appropriate washout periods can precipitate TdP and sudden cardiac arrest. Vigilant review of interacting QT‑prolonging agents, ECG surveillance, and electrolyte correction are essential. Severe QT prolongation may progress to polymorphic ventricular tachycardia (TdP), syncope, cardiac arrest, and death, which were observed across case series and cohort studies [3,4]. Early recognition and drug discontinuation usually reverse acquired QT prolongation and reduce TdP risk. Drug-induced QT prolongation is a common and potentially dangerous condition linked to TdP, with numerous implicated medications, necessitating awareness of mechanisms, risk factors, monitoring strategies, and preventive and therapeutic approaches [5].

We present a case of an elderly female patient who developed severe QT prolongation (QTc 626 ms) and polymorphic ventricular tachycardia following the administration of intravenous amiodarone while on chronic sotalol therapy, highlighting the critical importance of drug washout protocols and the life-saving role of overdrive pacing in managing drug-induced ventricular arrhythmias.

## Case presentation

An 88-year-old female patient with permanent atrial fibrillation, sick sinus syndrome status post dual-chamber pacemaker, heart failure with reduced ejection fraction (35-40%), hypertension, and hypothyroidism presented from a nursing facility with acute worsening shortness of breath, hypoxia, generalized weakness, and fatigue. Facility staff noted oxygen saturations dropping into the 80s, prompting activation of emergency medical services. Home medication included apixaban 2.5 mg twice daily, atorvastatin 20 once a day, levothyroxine 25 mcg, sotalol 80 mg twice daily, and lisinopril 20 mg twice a day. The last dose of sotalol 80 mg for her atrial fibrillation was taken within 24 hours of presentation.

On arrival, she was tachycardic (120-150 bpm, irregular), hypoxemic, and tachypneic. She was initially placed on 15 L via a non-rebreather mask and subsequently transitioned to bilevel positive airway pressure with a fraction of inspired oxygen (FiO_2_) of 40%, maintaining oxygen saturation above 95% with preserved mentation. She received breathing treatments for presumed chronic obstructive pulmonary disease exacerbation in the emergency department. Labs on admission showed leukocytosis, lactic acidosis, mildly elevated B-type natriuretic peptide, normal troponins, and electrolytes (Table 1). The initial EKG demonstrated atrial fibrillation with rapid ventricular response (RVR) (Figure 1A). Given persistent atrial fibrillation with RVR in the setting of heart failure and respiratory distress, the patient was subsequently admitted to the ICU for further management. Additionally, for severe sepsis due to hospital-acquired pneumonia (recently discharged), she was started on IV piperacillin-tazobactam and IV doxycycline, which are not associated with clinically significant QT prolongation.

**Table 1:** Laboratory values on admission Reference ranges are institution-dependent and provided for general guidance. Abnormal values are indicated as high or low based on adult reference ranges provided.

Parameter	Value	Reference Range	Interpretation
Hematology			
White blood cell (WBC)	15.50 × 10³/µL	4.0–11.0	High
Hemoglobin (Hgb)	13.2 g/dL	13.5–17.5	Normal
Platelet count	254 × 10³/µL	150–400	Normal
Chemistry			
Sodium	134 mmol/L	135–145	Low
Potassium	4.7 mmol/L	3.5–5.0	Normal
Chloride	100 mmol/L	98–106	Normal
CO₂ (bicarbonate)	18 mmol/L	22–29	Low
Calcium	9.6 mg/dL	8.5–10.5	Normal
Creatinine	1.2 mg/dL	0.6–1.3	Normal
Liver function tests			
Alanine aminotransferase (ALT)	61 U/L	7–56	High
Aspartate aminotransferase (AST)	57 U/L	10–40	High
Total bilirubin	0.5 mg/dL	0.1–1.2	Normal
Other tests			
C-reactive protein (CRP)	0.49 mg/dL	<1.0	Normal
Lactic acid	7.1 mmol/L	0.5–2.0	High
Magnesium	2.40 mg/dL	1.7–2.2	High
Phosphorus	5.7 mg/dL	2.5–4.5	High
Troponin I	16.9 pg/mL	8.7-18.7	Normal
B-type natriuretic peptide (BNP)	734 pg/mL	<100	High

**Figure 1:**
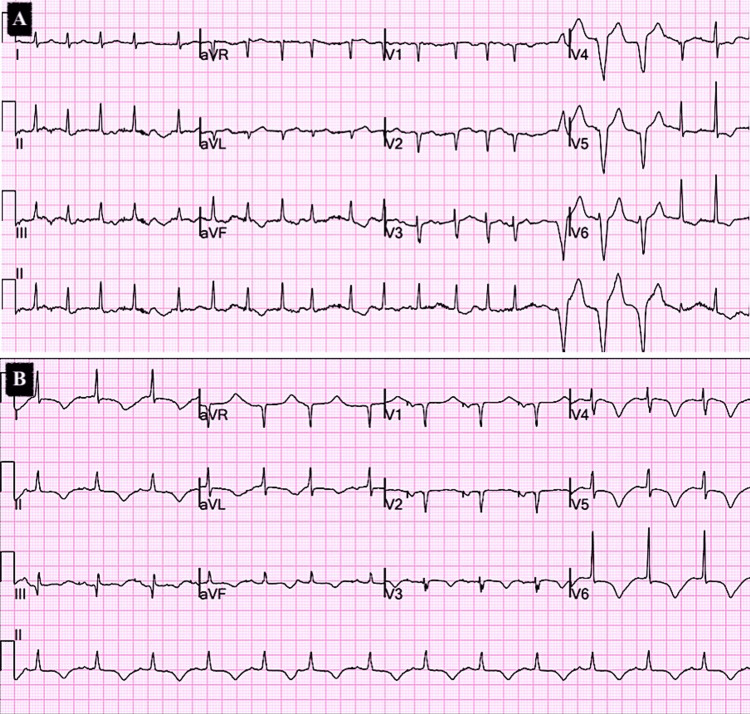
Electrocardiographic findings before and after amiodarone administration in a patient on chronic sotalol therapy. Panel A: Electrocardiogram at presentation showing atrial fibrillation with rapid ventricular response (heart rate of 129 beats per minute) with some V-paced rhythm in precordial leads prior to amiodarone administration. Panel B: Electrocardiogram 6 hours after intravenous amiodarone initiation, demonstrating atrial-paced rhythm with severe drug-induced QT prolongation. The corrected QT interval (QTc: Bazett formula, QTc = QT / √RR) is markedly prolonged at 626 ms. Note the deep, symmetric T-wave inversions across precordial leads V1–V6, which suggest drug-induced ventricular repolarization abnormalities.

Despite supportive care and intravenous metoprolol 5 mg bolus, atrial fibrillation with RVR persisted, prompting initiation of intravenous amiodarone (150 mg bolus followed by 1 mg/min infusion). Within 6 hours, she converted to an atrial-paced regular rhythm at 70 bpm, but repeat ECG showed concerning findings (Figure 1B). ECG demonstrates delayed ventricular repolarization due to combined class III antiarrhythmic effects, leading to marked QT prolongation.

Amiodarone was discontinued immediately. Intravenous magnesium sulfate (2 grams over 15 minutes) was given emergently, as it suppresses early after-depolarizations. A critical intervention with overdrive pacing was initiated, with the pacing rate increased from 60 to 80 beats per minute. This intervention resulted in immediate suppression of the ventricular ectopy and NSVTs. Pacemaker interrogation revealed multiple episodes of NSVT. The patient was monitored in the ICU with continuous telemetry. Over the subsequent 24-48 hours, ECGs showed a progressive decrease in the QTc interval.

However, the patient's condition worsened with multiorgan failure due to septic shock secondary to hospital-acquired pneumonia, requiring hemodialysis. The patient and family opted for comfort-focused care, and the patient was subsequently discharged to a nursing facility.

## Discussion

This case illustrates the potentially catastrophic consequences of combining class III antiarrhythmic agents without appropriate washout periods and highlights several critical clinical principles in the management of atrial fibrillation and drug-induced QT prolongation.

QTc interval is considered prolonged when it is >460 ms in women (>450 ms in men), with >500 ms linked to a higher risk of TdP. Figure 1B demonstrates QT prolongation (QTc: 626 ms) from overlapping class III antiarrhythmic therapy, critically increasing risk for TdP. Deep, symmetric T-wave inversions across precordial leads (V1-V6) are suggestive of drug-induced repolarization abnormalities. Both sotalol and amiodarone block IKr potassium channels, prolonging ventricular action potential duration and QT interval. Without adequate washout (at least three half-lives; 36 hours for sotalol), their effects are additive [1]. Sotalol carries a much higher risk of QT prolongation compared to amiodarone; therefore, guidelines recommend that initiation or dose escalation of sotalol requires admission for at least three days in a facility capable of calculating creatinine clearance, providing continuous ECG monitoring, and delivering advanced cardiac resuscitation if needed [6].

Additionally, patients with sepsis are more susceptible to drug-induced QT prolongation. In critically ill or hemodynamically compromised patients, even therapeutic doses of QT-prolonging drugs may precipitate life-threatening arrhythmias. Emerging evidence also highlights the role of systemic illness in modulating arrhythmia risk. Pamporis et al. recently reviewed the pathophysiology of sepsis-induced cardiomyopathy and cardiac arrhythmias, demonstrating that systemic inflammation, myocardial ischemia, autonomic dysfunction, and electrolyte disturbances collectively create a vulnerable substrate for QT prolongation and ventricular arrhythmias [7].

Device-based overdrive pacing prevents bradycardia and pauses dependent arrhythmias by shortening action potential duration and QT interval, thereby reducing the risk of TdP [2]. Electrolytes should be optimized with continuous telemetry monitoring. Tötterman et al. reported one of the earliest clinical series demonstrating the efficacy of overdrive pacing for sotalol-induced TdP, showing immediate suppression of ventricular arrhythmias with temporary overdrive pacing [8]. This experience has been replicated across multiple case series and is now incorporated into consensus guidelines for the management of drug-induced QT prolongation [1,2]. In our patient, increasing the pacing rate from 60 to 80 beats per minute was sufficient to suppress ventricular ectopy and prevent progression to sustained TdP.

This case reinforces several evidence-based strategies for preventing drug-induced TdP. First, baseline ECG assessment with QTc measurement is mandatory before initiating any QT-prolonging antiarrhythmic drug [1,2]. Patients with QTc >500 ms or QTc prolongation >60 ms from baseline during therapy are at markedly elevated risk of Tdp, and thus prompt action is needed, including alternate pharmacotherapy [2]. Second, when transitioning between class III antiarrhythmic drugs, appropriate washout periods must be observed. Third, vigilant monitoring is essential during the initiation and titration of antiarrhythmic drugs. Finally, a comprehensive medication reconciliation must identify all potential sources of QT prolongation, including non-cardiac drugs (antibiotics, antipsychotics, antiemetics) that may contribute to additive risk.

## Conclusions

This case of severe QT prolongation and polymorphic ventricular tachycardia following dual class III antiarrhythmic therapy serves as a critical reminder of the significant proarrhythmic potential inherent to rhythm-control strategies in atrial fibrillation. The concurrent administration of sotalol and amiodarone without appropriate washout created a pharmacologic storm, resulting in life-threatening ventricular arrhythmias in a vulnerable elderly patient. Patient-specific risk factors including advanced age, female sex, structural heart disease, electrolyte disturbances, and systemic illness must be systematically assessed and mitigated before initiating QT-prolonging drugs. Immediate discontinuation of antiarrhythmic agents, intravenous magnesium, and device-based overdrive pacing are critical to prevent TdP.

From a broader clinical perspective, this case underscores the imperative for a cautious, evidence-based approach to antiarrhythmic therapy. The enthusiasm for aggressive rhythm control must be tempered by a realistic appraisal of proarrhythmic risk, particularly in elderly patients with multiple comorbidities. Mandatory baseline and serial ECG monitoring, aggressive electrolyte repletion, comprehensive medication reconciliation, and strict adherence to washout protocols between antiarrhythmic agents represent evidence-based strategies to minimize the risk of catastrophic proarrhythmic events.
